# Surgical management of submacular hemorrhage due to n-AMD: a comparison of three surgical methods

**DOI:** 10.1186/s40942-020-00228-x

**Published:** 2020-07-02

**Authors:** Carsten Grohmann, Spyridon Dimopoulos, Karl Ulrich Bartz-Schmidt, Philipp Schindler, Toam Katz, Martin S. Spitzer, Christos Skevas

**Affiliations:** 1 Department of Ophthalmology, University Medical Center Hamburg-Eppendorf (UKE), Hamburg, Germany; 2 Department of Ophthalmology, Eberhard Karls University Medical Center, Tübingen, Germany

**Keywords:** Acute submacular hemorrhage (ASH), Neovascular age related macular degeneration (n-AMD), Choroidal neovascularization (CNV), Vascular endothelial growth factor (VEGF), Retinal pigment epithelium (RPE), Central foveal thickness (CFT), Pars plana vitrectomy (PPV), Recombinant tissue plasminogen activator (rtPA)

## Abstract

**Background:**

To compare and assess the efficacy of three surgical methods for the treatment of acute submacular hemorrhage (ASH): pneumatic displacement with C2F6, in combination with intravitreal injection of bevacizumab and rtPA, pars plana vitrectomy (PPV) with intravitreal injection of gas (C2F6), bevacizumab and subretinal injection of recombinant tissue plasminogen activator (rtPA), pars plana vitrectomy (PPV) with intravitreal injection of gas (C2F6), bevacizumab and intravitreal injection of recombinant tissue plasminogen activator (rtPA).

**Methods:**

The study included 85 patients with ASH. In the group without PPV (A), patients were treated with intravitreal injection of C2F6, bevacizumab and rtPA. In the second and third group, patients underwent a PPV, intravitreal injection of bevacizumab, pneumatic displacement with C2F6 and a subretinal (B) or intravitreal (C) injection of recombinant tissue plasminogen activator (rt PA).

**Results:**

In group A, mean BCVA increased from preop logMAR 1.41 to 1.05, in group B from 1.46 to 1.28 and in group C from 1.63 to 1.33. In group A, CFT changed from 764 ± 340 μm at time point 0 to 246 ± 153 μm at time point 1, in group B from 987 ± 441 μm to 294 ± 166 μm and in group C from 642 ± 322 μm to 418 ± 364 μm. Patients had an average of 5 injections after surgery.

**Conclusion:**

Our study demonstrates that the three methods are equally effective in improving the morphology and the BCVA of patients with ASH.

## Background

A devastating complication of a n-AMD is an acute submacular hemorrhage (ASH) [1]

The localization of the hemorrhage may vary between the neurosensory retina and the RPE or beneath the RPE. The harmful consequences on the retina can be attributed to a limited passage of nutrients, the shrinkage of the outer retinal layers due to clot formation and the release of toxic substances like iron, hemosiderin and fibrin. Toxic effects of subretinal blood can be demonstrated 24 h after hemorrhage [2–6].

Prognosis of an untreated choroidal neovascularization (CNV), which is also complicated by an ASH, is poor and can lead to permanent vision loss. The underlying CNV may progress, and the resolution of the hemorrhage leads to a macular scar formation [1, 7–10].

Other causes that can lead to an ASH are high myopia, trauma and arterial macroaneurysm. Duration of the bleeding, size of the hemorrhage and thickness of the blood clot, among other factors, can play a decisive role in the visual prognosis [11].

To date, there is no standardized treatment protocol for ASH due to n-AMD, but a variety of therapeutic approaches have been described. The common goal of all those treatment options is the rapid displacement of submacular/subretinal blood from the fovea in order to prevent damage to the photoreceptors [11]. These include intravitreal administration of gas, intravitreal or subretinal application of recombinant tissue plasminogen activator (rt-PA), intravitreal injection of anti-VEGF substances, pars plana vitrectomy (PPV) and subretinal clot removal [12].

The evolution of these new treatment modalities gave hope to many patients whose prognosis might be dire and paved the way for a discussion among surgeons and authors about which surgical therapy is more effective and advantageous for the patients. The purpose of this study is to address this question. Principal outcome measures were displacement of hemorrhage, complications, best corrected visual acuity (BCVA) and central foveal thickness (CFT).

## Methods

Our study was retrospective in nature and included a total number of 85 patients treated at the university clinic Eppendorf in Hamburg and Eberhard Carls in Tübingen, Germany. The investigation adhered to the tenets of the declaration of Helsinki. The etiology of the ASH in our patients was n-AMD in all cases. Exclusion criteria were massive submacular hemorrhages extending beyond the equator, RPE detachments and sub-RPE bleedings, macular scar, polypoidal choroidal vasculopathy and other etiology for ASH like high myopia, trauma and arterial macroaneurysm. Before initiating treatment, all strategies were thoroughly discussed with the patients, along with the possibility of no treatment as none of the offered treatment modalities are clearly evidence based. All patients rejected the no-treatment option.

Our study included three groups of surgical treatment.

In the first group (group A) in which no PPV was performed, patients were treated with intravitreal injection of C2F6 (Hexafluorethan-16%) gas, 1.25 mg of bevacizumab and recombinant tissue plasminogen activator (rtPA) (20 µg (0.1 ml). [Rt-PA (Actilyse^®^, Boehringer Ingelheim, Ingelheim, Germany)]. Group A included 32 patients. The mean extent of the hemorrhage in this group was 4.4 ± 1.8 PD [papillary diameter (PD)].

In the second and third group (groups B and C), patients underwent a standard three-port PPV (23G), intravitreal injection of 1.25 mg bevacizumab, pneumatic displacement with C2F6 (Hexafluorethan-16%) gas and injection of rt PA. The difference between groups B and C is that in group B patients received rt PA subretinally (20 µg (0.1 ml)) and in group C intravitreally (20 µg (0.1 ml)). The injection of rt PA into the subretinal space was performed utilizing a 41 G transretinal needle. A subretinal bleb was created in order to bathe the entire clot and to allow for optimal displacement of the hemorrhage. Following rtPA injection, air-fluid and air-gas exchange was performed. The mean PD of the hemorrhage in group B was 4.14 ± 1.3 PD and in group C 4.68 ± 2.8 PD.

Group B included 42 patients, and group C included 11 patients.

In all treatment groups, patients were instructed to keep a prone position for at least 7 days postoperatively. All patients were advised to attend our institution for follow-up examinations.

Principal outcome measures were displacement of hemorrhage, BCVA, CFT and complications (intraoperative and postoperative). Preoperative and postoperative evaluation included a complete ocular examination comprised of a slitlamp examination (anterior and posterior chamber evaluation), BCVA, intraocular pressure measurement, CFT with spectral domain optical coherence tomography [SD-OCT (Heidelberg Spectralis; Heidelberg Engineering, Heidelberg, Germany)] and fluorescein angiography (FLA).

The average interval from the onset of symptoms to surgery was 9.1 ± 4.6 days. Surgery was performed the day after preoperative examination (time point 0), and the last evaluation follow-up was 6 months after surgery (time point 1).

All patients fully acknowledged the potential risks and all possible postoperative consequences before surgery. Written informed consent was obtained from each patient and his or her family members after discussion of the procedure.

For statistical analyses, BCVA was analyzed on a logarithm of the minimum angle of resolution (logMAR) scale. In the current study, visual acuity of counting fingers and hand motion was converted as 2.0 and 3.0 logMAR, respectively.

### Statistical analysis

All data were expressed as mean ± standard deviation and compared using the Wilcoxon Signed-Rank Test, ANOVA-type statistics and the pairwise Mann–Whitney test. Statistical analyses were performed using SPSS 19.0 software. Statistical significance was considered when p < 0.05.

## Results

Our study included 32 men and 53 women. The mean patient age was 85.36 years (range 53–102 years). The mean follow-up period was 6.1 months (range 5–7 months).

### Postoperative visual outcome

In group A, mean BCVA was preop logMAR 1.41[(SD) = 0.48] and postop 1.05 [(SD = 0.52)]. BCVA improved in 22, remained unchanged in 3 and decreased in 7 patients.

In group B, mean BCVA was preop logMAR 1.46 [(SD) = 0.54] and postop 1.28 [(SD = 0.61)]. BCVA improved in 23, remained unchanged in 7 and decreased in 12 patients.

In group C, mean BCVA was preop logMAR 1.63 [(SD) = 0.53] and postop 1.33 [(SD = 0.59)].

BCVA improved in 6, remained unchanged in 2 and decreased in 3 patients.

Table 1 shows the descriptive statistics for the BCVA and the macular thickness at the preoperative timepoint. Table 2 shows the descriptive statistics at the timepoint of the 6 months follow-up. The statistics for the visual outcome can be found in Table 3. BCVA did not differ significantly between the three groups, neither at the preoperative nor at the postoperative time point, which shows that BCVA improved significantly only in groups C and A, with p = 0.013 and p = 0.001 respectively. Ranks and Relative Effects confirm this result: ranks in group B are very similar at each time point, and RTEs (relative treatment effects) are all around 0.5. No main effect of one of the three groups was observed (the BCVA in all three groups is similar); there is overall significant improvement in BCVA (We saw, however, that this overall effect is mainly due to the improvements in groups A and C.). There is no interaction between time and groups.

**Table 1:** Descriptive statistics for Visus and macular thickness (in µm) preoperative

Groups	Type	N	Mean	SD	Lower	Upper	Median	Q25	Q75
A	Visus	34	1.41	0.48	1.26	1.57	1.3	1.02	2.03
A	Macular	32	764	340	656	886	715	535	935
B	Visus	47	1.46	0.54	1.30	1.61	1.4	1.00	2.10
B	Macular	42	987	441	857	1120	890	692	1200
C	Visus	23	1.63	0.53	1.40	1.83	1.8	1.30	2.10
C	Macular	11	642	322	473	845	599	408	768

**Table 2:** Descriptive statistics for Visus and macular thickness (in µm) at the time of follow up (6 months, postop)

Groups	Time	N	Mean	SD	Lower	Upper	Median	Q25	Q75
A	Visus	34	1.05	0.52	0.88	1.22	1.0	0.62	1.53
A	Macular	32	246	135	204	297	208	189	310
B	Visus	47	1.28	0.61	1.11	1.46	1.3	0.90	1.70
B	Macular	42	294	166	246	343	281	170	388
C	Visus	23	1.33	0.59	1.09	1.57	1.2	1.00	1.90
C	Macular	11	418	365	250	663	266	252	444

**Table 3:** Analytical statistics (Kruskal–Wallis test, Wilcoxon signed rank test, ANOVA-type statistics and rank statistics) for Visus preoperative and at the time of follow up (6 months, postop)

Groups	Time	DF	V/Stat.	Chi^2^	p	Rank means	N	RTE	type
A	–	–	389.5	–	0.013	–	–	–	Wilcox
B	–	–	527.0	–	0.057	–	–	–	Wilcox
C	–	–	129.5	–	0.013	–	–	–	Wilcox
–	Preop	2	–	2.41	0.300	–	–	–	Kruskal
–	Postop	2	–	4.13	0.127	–	–	–	Kruskal
Groups	–	1.81	2.20	–	0.116	–	–	–	ANOVA
–	Time	1.00	21.54	–	0.000	–	–	–	ANOVA
Groups	Time	2.00	0.84	–	0.432	–	–	–	ANOVA
A	–	–	–	–	–	92	68	0.44	Rank
B	–	–	–	–	–	107	94	0.51	Rank
C	–	–	–	–	–	118	46	0.56	Rank
–	Preop	–	–	–	–	120	104	0.58	Rank
–	Postop	–	–	–	–	91	104	0.44	Rank
A	Preop	–	–	–	–	111	34	0.53	Rank
A	Postop	–	–	–	–	73	34	0.35	Rank
B	Preop	–	–	–	–	116	47	0.55	Rank
B	Postop	–	–	–	–	98	47	0.47	Rank
C	Preop	–	–	–	–	133	23	0.64	Rank
C	Postop	–	–	–	–	102	23	0.49	Rank

The data suggests that the BCVA among the three groups is not statistically significant at time point 0 (pre-op) and time point 1 (6 months post-op).

#### Complications

There were no intraoperative complications. There were two retinal detachments (one in the group with intravitreal injection and one in the group of the PPV with intravitreal injection of rtPA). There were no cases of infectious endophthalmitis.

### Anatomical outcome

In group A, CFT changed from 764 ± 340 μm at baseline to 246 ± 153 μm at 6 months.

In group B, CFT changed from 987 ± 441 μm at baseline to 294 ± 166 μm at 6 months.

In group C, CFT changed from 642 ± 322 μm at baseline to 418 ± 364 μm at 6 months.

Patients, on average, had 5 injections after surgery, given in monthly intervals after surgery (0 to 9 injections; bevacizumab). The re-treatment criteria were signs of CNV activity seen on OCT or angiography. Tables 1 and 2 show the descriptive statistics for BCVA. In group A, 4 out of 32 patients did not receive intravitreal injections after surgery. In group B, 7 out of 42 patients, and in group C only 3 out of 11 did not receive intravitreal injections after surgery. Most patients received a series of three anti-VEGF injections every 4 weeks followed by a treat-and-extend scheme.

In Table 4, the analytical statistics of the different groups for the macular thickness are shown. Kruskal–Wallis test confirmed that there were significant differences between the groups at time point 0 but not at time point 1. There was only a significant difference between groups B and C. This rank-based approach tests the main effects of groups and time and allows for the testing of interaction terms. First, let us look at mean ranks and the relative effects associated with groups, occasions and their interactions.

**Table 4:** Analytical statistics (Kruskal–Wallis test, Wilcoxon signed rank test, ANOVA-type statistics and rank statistics, interaction contrasts and pairwise Mann–Whitney-Test) for macular thickness preoperative and at the time of follow up (6 months, postop)

Groups	Time	DF	V/Stat.	Chi^2^	p	Rank means	N	RTE	Type
A	–	–	526	–	0.000	–	–	–	Wilcox
B	–	–	894	–	0.000	–	–	–	Wilcox
C	–	–	50	–	0.147	–	–	–	Wilcox
–	Preop	2	–	9.46	0.009	–	–	–	Kruskal
–	Postop	2	–	3.28	0.194	–	–	–	Kruskal
Groups	–	1.53	1.30	–	0.267	–	–	–	ANOVA
–	Time	1.00	103.68	–	0.000	–	–	–	ANOVA
Groups	Time	1.40	4.72	–	0.018	–	–	–	ANOVA
A	–	–	–	–	–	79	64	0.46	Rank
B	–	–	–	–	–	91	84	0.53	Rank
C	–	–	–	–	–	84	22	0.49	Rank
–	Preop	–	–	–	–	118	85	0.69	Rank
–	Postop	–	–	–	–	52	85	0.30	Rank
A	Preop	–	–	–	–	117	32	0.68	Rank
A	Postop	–	–	–	–	41	32	0.24	Rank
B	Preop	–	–	–	–	133	42	0.78	Rank
B	Postop	–	–	–	–	49	42	0.29	Rank
C	Preop	–	–	–	–	103	11	0.61	Rank
C	Postop	–	–	–	–	41	11	0.38	Rank
A:B	–	–	–	–	0.079	–	–	–	Pairwise
A:C	–	–	–	–	0.587	–	–	–	Pairwise
B:C	–	–	–	–	0.028	–	–	–	Pairwise
B:A	Pre:Post	–	–	–	1.000	–	–	–	Interact
C:B	Pre:Post	–	–	–	0.51	–	–	–	Interact
C:A	Pre:Post	–	–	–	0.081	–	–	–	Interact

We see the ranks for group C, followed by group B and group A. So, taking all measurements together (both occasions), group C had higher values compared to the other two groups. These differences were not large.

Indeed, relative effects were all around 0.5. For instance, the relative effect (RTE) 0.53 for group B (PPV with rt PA subretinal) can be interpreted as follows: a randomly chosen observation from the whole dataset results in a smaller value than a randomly chosen observation from group B (PPV with rt PA subretinal) with an estimated probability of 53%. So, nearly 50% of the patients have lower or higher values on macular thickness than a randomly selected patient from group B (PPV with rt PA subretinal). In other words, there is no difference between the patients from this group and those from the others. The effect of groups is not statistically significant, p = 0.267. On the other hand, the main effect of time is highly significant, p < 0.001. From Table 4 we see that the probability that the randomly chosen observation from the whole dataset will have a smaller value on retinal thickness than a randomly selected patient on the second occasion is only 30%. This means that there is a significant decrease of macular thickness from time point 0 to time point 1, and this tells us that there is overall a significant interaction between groups and time points. We can fit separate ANOVA-type models to investigate the interactions between groups and measurement time. We see that while there was an overall significant interaction, the pairwise comparisons lacked a statistically significant effect (at a 0.05 level). Thus, the interaction should be confirmed by sampling more data.

The mean interval from the onset of symptoms to surgery was 9.1 ± 4.6 days, and the average size of the subretinal hemorrhage was 4.5 ± 1.0 disc diameters. The submacular hemorrhage was displaced from the foveal area in all eyes after 1 week.

## Discussion

ASH is a common manifestation of n-AMD. It is associated with sudden visual loss, and the prognosis of ASH is often poor. The reduced visual outcome is caused by retinal toxicity of subretinal blood, which includes limited diffusion of nutrients and oxygen, damaging photoreceptor outer segments due to clot contraction and release of toxic materials. The functional outcome may also be influenced by the duration and the size of submacular hemorrhage. Time is believed to be of essence in treating ASH, and this is the reason why a prompt evacuation of ASH is supported by many investigators. We included only patients with hemorrhages of ≤ 14 days because older ASH are associated with significantly worse functional outcomes [1, 5, 6, 11, 13, 14].

The correct strategy for massive ASH secondary to CNV is still under debate. Up to now there have been no standard-of-care recommendations but a variety of therapeutic approaches have been described including intravitreal administration of gas, intravitreal or subretinal application of rt-PA, intravitreal injection of anti-VEGF substances, vitrectomy and subretinal clot removal [11, 12].

The aim of our retrospective study was a comparison of three surgical treatment options that are available today and to try to elucidate the most effective of them. Nevertheless, this study is a retrospective one without a control group. Between the surgical procedure in the hospital and the follow-up appointment 6 months after surgery, a number of patients failed to attend their follow-up injections: a number of them received their anti-VEGF upload and, if needed, additional intravitreal injections elsewhere.

More than 30 years have elapsed since the early 1990s when rtPA was introduced to facilitate clot liquefaction. RtPA is a serine protease, cleaving plasminogen into active plasmin, which in turn degrades the fibrin matrix constituting blood clots. In addition, other proteins such as hepatocyte growth factor or platelet derived growth factor are also cleaved by rtPA [15, 16]. The use of rtPA has been reported as a surgical adjunct administered both intravitreally and in the subretinal space [17, 18]. Lewis et al. reported a successful liquefaction of up to 70–80% of the cases [19, 20].

One of the most common therapeutic approaches for ASH is intravitreal injection of rt-PA and gas. This method is minimally invasive and requires less technical equipment and less surgical skills. Heriot was the first to describe a successful pneumatic displacement of ASH with intravitreal injection of rtPA [21, 22]. A number of cases and publications demonstrated the effectiveness of the intravitreal application of rt-PA in combination with gas [23–25]. Chen et al. reported success in 60–100% of patients treated with rt PA and gas [26, 27]. Injection gas or rtPA in non vitrectomized eyes can lead to severe complications like dense vitreous hemorrhage, retinal breaks or even retinal detachments [28–30]. Our study presented two cases of retinal detachment, one of them being in the group of pneumatic displacement without vitrectomy.

VEGF is widely considered the main growth factor leading to increased angiogenesis and vascular leakage [31]. Without anti-VEGF therapy, visual acuity frequently deteriorates because of progression of the underlying CNV. The advent of anti-VEGF pharmacotherapy revolutionized the management of n-AMD. Even though they are not always effective in restoring or improving visual acuity (VA) when a large ASH is present [32], a small number of clinical studies with small study populations showed encouraging results [33–38].

On the one side, the application of rt-PA and gas can help to prevent a toxic effect of ASH by effective displacement from the fovea, and on the other side, the anti-VEGF agents can potentially prevent CNV progression. Our study group and various authors have reported positive results [12, 13, 25, 39, 40].

With the introduction of small gauge, PPV removal of the vitreous became less invasive [41]. The surgical extraction of the neovascular complex after an ASH was proposed in the 1980s. The submacular blood clot was removed after performing a retinotomy. The results were not promising due to poor visual acuity results (atrophy of the RPE) and frequent complications, e.g. retinal detachment, and the surgical technique was abandoned soon afterwards [42–44]. The results of the Submacular Surgery Trial (SST) showed no clear benefit of surgical removal of AMD-associated hemorrhagic CNV lesions [45, 46].

Delivery of rt-PA to the subretinal space can be ensured via subretinal injection, but there are some reservations concerning subretinal injection of rt PA. Surgical manipulations including creation of a retinotomy and injections into the subretinal space can induce considerable damage to the RPE, which leads to the gradual degeneration of photoreceptors and progressive visual impairment [17]. PPV with a subretinal or intravitreal injection of rt-PA and a gas tamponade can effectively displace a submacular hemorrhage away from the fovea. A number of authors reported positive anatomical and functional results [12, 18, 45, 47–50]. Both minimally invasive methods were, in later studies, combined with anti-VEGF therapy [50], allowing for simultaneous treatment of the causative CNV [27]. Hillenkamp and associates compared the effects of an intravitreal or subretinal injection of rt-PA and vitrectomy and gas tamponade in eyes with ASH. They reported that these procedures were more effective than vitrectomy with intravitreal injection of rt-PA and gas tamponade in displacing a submacular hemorrhage. The incidence of postoperative complications consisting of vitreous hemorrhage, retinal detachment and recurrent submacular hemorrhage was higher after a subretinal injection than after intravitreal injection of rt-PA [12]. Even though our study showed that both intravitreal and subretinal injections of rt PA were advantageous for the patient, neither of them proved to be more effective.

In order to address the critical issue of intraoperative and postoperative complications, Cho et al. recommend the less invasive treatment option of anti-VEGF injections as the most effective strategy against ASH associated with AMD. Their study showed no significant therapeutic difference between an anti-VEGF monotherapy and anti-VEGF combined with pneumatic displacement [51].

A new third-generation thrombolytic agent (tenecteplase-TNK), which is a variant of tPA, has been produced by recombinant DNA technology [52]. With a longer half-life and greater fibrin specificity, TNK is potentially more efficacious in thrombus dissolution [53]. A number of studies have shown no retinal toxicity after subretinal TNK injection and positive results after intravitreal injection in humans [54].

Our study excluded PED cases. Fibrovascular PEDs may be difficult to treat, but even these eyes can gain vision after anti-VEGF therapy. A RPE tear may develop in 15% to 20% of eyes with PEDs after anti-VEGF therapy; however, vision may stabilize with continued therapy. Atrophy may complicate eyes with PED and nAMD after anti-VEGF therapy, especially in association with complete PED resolution [55, 56].

## Conclusions

The management of ASH is controversial, and a standardized management protocol still does not exist. Various factors can determine the final outcome and the prognosis of VA. Age, size, duration of the hemorrhage and patients’ health status are significant factors. As discussed above, a subretinal bleeding can have devastating consequences on the health and integrity of the photoreceptors and the RPE so that even a prompt evacuation of the ASH in combination with anti-VEGF agents, which constitute the mainstay of CNV treatment, could not significantly change and improve the VA. We have demonstrated that the three well-known and established treatment methods help to improve morphology and BCVA, but none of them had any clear advantage over the others. The continuation of the anti-VEGF regimen is very important to further suppress the CNV formation and activity. Patients health status and treatment costs have to be taken into account. In light of our findings, we can support pneumatic displacement combined with intravitreal anti-VEGF as a treatment option due to its minimally invasive character and non-inferior anatomical and functional results.

Our study has several limitations, including its retrospective nature and the small number of patients in group C. Moreover, there was no control study group without any treatment.

## Data Availability

The datasets used and analyzed during the current study are available from the corresponding author on reasonable request.
